# The Effects of Probiotics, Prebiotics and Synbiotics in Non-Alcoholic Fat Liver Disease (NAFLD) and Non-Alcoholic Steatohepatitis (NASH): A Systematic Review

**DOI:** 10.3390/ijms23158805

**Published:** 2022-08-08

**Authors:** Rodrigo Zamignan Carpi, Sandra M. Barbalho, Katia Portero Sloan, Lucas Fornari Laurindo, Heron Fernando Gonzaga, Paulo Cesar Grippa, Tereza L. Menegucci Zutin, Raul J. S. Girio, Cláudia Sampaio Fonseca Repetti, Cláudia Rucco Penteado Detregiachi, Patrícia C. Santos Bueno, Eliana de Souza Bastos Mazuqueli Pereira, Ricardo de Alvares Goulart, Jesselina Francisco dos Santos Haber

**Affiliations:** 1 Department of Biochemistry and Pharmacology, School of Medicine, University of Marília (UNIMAR), Avenida Hygino Muzzy Filho, 1001, Marilia 17525-902, SP, Brazil; 2 Postgraduate Program in Structural and Functional Interactions in Rehabilitation, University of Marília (UNIMAR), Marilia 17525-902, SP, Brazil; 3 School of Food and Technology of Marilia (FATEC), Marilia 17506-000, SP, Brazil; 4 Texas Institute for Kidney and Endocrine Disorders, Lufkin, TX 75904, USA; 5 Department of Animal Sciences, School of Veterinary Medicine, University of Marília (UNIMAR), Avenida Hygino Muzzy Filho, 1001, Marilia 17525-902, SP, Brazil; 6 Department of Biochemistry, School of Dentistry, University of Marília (UNIMAR), Avenida Hygino Muzzy Filho, 1001, Marilia 17525-902, SP, Brazil

**Keywords:** non-alcoholic fatty liver disease (NAFLD), non-alcoholic steatohepatitis (NASH), prebiotic, probiotic, symbiotic, microbiota

## Abstract

Modifications in the microbiota caused by environmental and genetic reasons can unbalance the intestinal homeostasis, deregulating the host’s metabolism and immune system, intensifying the risk factors for the development and aggravation of non-alcoholic fat liver disease (NAFLD). The use of probiotics, prebiotics and synbiotics have been considered a potential and promising strategy to regulate the gut microbiota and produce beneficial effects in patients with liver conditions. For this reason, this review aimed to evaluate the effectiveness of probiotics, prebiotics, and symbiotics in patients with NAFLD and NASH. Pubmed, Embase, and Cochrane databases were consulted, and PRISMA (Preferred Reporting Items for Systematic Reviews and Meta-Analysis) guidelines were followed. The clinical trials used in this study demonstrated that gut microbiota interventions could improve a wide range of markers of inflammation, glycemia, insulin resistance, dyslipidemia, obesity, liver injury (decrease of hepatic enzymes and steatosis and fibrosis). Although microbiota modulators do not play a healing role, they can work as an important adjunct therapy in pathological processes involving NAFLD and its spectrums, either by improving the intestinal barrier or by preventing the formation of toxic metabolites for the liver or by acting on the immune system.

## 1. Introduction

The accelerated industrialization and urbanization processes have evidenced the exponential increase in ultra-processed food consumption and the predominance of a sedentary lifestyle. This setting has developed an epidemic of obesity, dyslipidemia, type 2 diabetes mellitus (DM2), and Metabolic Syndrome (MS) that are associated with the development of non-alcoholic fatty liver disease (NAFLD) that is the most prevalent chronic liver disease worldwide [1,2,3]. The global occurrence of NAFLD is 25.24%, and the highest prevalence rate of the disease is found in the Middle East (31.79%), followed by South America (30.45%), Asia (27.37%), North America (24.13%), Europe (23.71%), and Africa (13.48%) [1,4].

NAFLD is a term that encompasses a cluster of disorders related to the macrovesicular accumulation of triglycerides within hepatocytes higher than 5%, thus causing steatosis. This process occurs due to a mismatch of regulatory mechanisms involved in lipid metabolism. Some patients develop a more aggressive subtype of the disease characterized by lobular inflammation plus hepatic balloon degeneration, with or without fibrosis, known as non-alcoholic steatohepatitis (NASH) [5,6,7].

Approximately 22.5% of NASH patients will develop hepatocellular carcinoma, and 20% will develop cirrhosis [8,9,10,11]. This progressive feature makes NASH the second most prevalent cause of liver transplantation in the USA [12]. Besides, NAFLD has an intense correlation to cardiovascular disease (CVD). A meta-analysis conducted by Haddad et al. showed that the prevalence of cardiovascular events in patients with NAFLD (14.9%) was more than two-fold compared to patients without NAFLD (6.2%) [13].

The burden of NAFLD is increasing worldwide, and its complications are severe. Numerous therapies have emerged to treat or slow the disease’s advance. Some studies have recently shown favourable results with interventions carried out in the intestine that reflect improvements in the liver, such as the bio modulation of intestinal microbiota [14,15,16,17,18]. The use of probiotics, prebiotics and synbiotics has been considered a potential and promising strategy to regulate the gut microbiota [19,20]. The intestinal microbiota is an intense and dynamic ambient whose composition continually changes. These alterations, caused by environmental and genetic reasons, can unbalance the intestinal homeostasis, deregulating the host’s metabolism and immune system, intensifying the risk factors for the development and aggravation of NAFLD [15,21]. 

Some reviews investigated the effects of the use of prebiotics and probiotics in NAFLD patients, but most evaluated the effectiveness only on hepatic enzymes [22,23]. Other reviews evaluated the effectiveness of prebiotics or probiotics alone [24,25,26,27]. Only two reviews compared the effects of probiotics, prebiotics and synbiotics on liver enzymes, but not on other risk factors associated with NAFDL and NASH [23,28]. Souza et al. [28] also performed a review comparing the effects of probiotics, prebiotics and synbiotics on NAFLD. Nevertheless, only four trials were included in the review. For these reasons, this systematic review aims to evaluate the effectiveness of probiotics, prebiotics and synbiotics in the management of NAFLD and NASH. To the best of our knowledge, this is the first review to show the effects of probiotics, prebiotics and synbiotics both in NAFLD and NASH.

### 1.1. NAFLD and NASH

NAFLD represents a clinicopathological spectrum of liver diseases extending from isolated steatosis fibrosis to cirrhosis and related to hepatocellular carcinoma development. It is defined as the development of steatosis in more than 5% of hepatocytes identified either histologically or radiologically, in the absence of secondary causes such as significant alcohol consumption (<30 g/day for man and <20 g/day for woman), hereditary liver diseases, or viral hepatitis [29]. This disease is comprised of two main entities: NAFLD and NASH. Histologically, the first includes any case characterized by steatosis with minimal or absent lobular inflammation. The second constitutes a more progressive form, characterized by balloon hepatocyte degeneration and diffuse lobular inflammation with or without fibrosis [5]. An increased risk of cirrhosis, hepatocellular carcinoma, and liver-related mortality is associated with NASH, especially when fibrosis is already present. In advanced stages of fibrosis, the mortality rate increases exponentially [30,31].

Although NASH may be suspected in the case of fatty liver and elevated liver enzymes, liver biopsy with histological examination is the only diagnostic method. On the other hand, the diagnosis of NAFLD may be made either by histological examination or by imaging studies that can detect more than 5% of hepatic steatosis. In this sense, ultrasound (US) and computed tomography (CT) are capable of detecting steatosis involving 20% of hepatocytes, and magnetic resonance imaging (MRI) is capable of detecting stenosis in 5% [32,33].

The NAFLD is currently recognized as a hepatic manifestation of MS [4,34] which shares a common pathogenic pathway in insulin resistance. The pathophysiology involves an imbalance between lipid acquisition, mitochondrial fatty acid oxidation, and its export as part of the very-low-density lipoprotein (VLDL) molecule that generates hepatic steatosis [35]. In this way, it is clear that the criteria for MS (dyslipidemia, hyperglycemia, central adiposity, and hypertension) and weight gain will be considered risk factors strongly related to the disease [36]. 

In NASH, the two-hit proposal has been used for years to explain the pathophysiology of the disease. The first involves insulin resistance that will cause steatosis, and the second is associated with the inflammatory process generated by lipid oxidation. Nevertheless, both hits are insufficient to explain the disease. Thus, a multi-hit theory was proposed, including (besides the phenomena of the old theory) lipotoxicity caused by the accumulation of free fatty acids, cholesterol, and triglycerides, Kupffer cell activation, myeloid cell recruitment, gut microbiota dysfunction, genetic factors, and diet [35,37,38]. 

**Figure 1:**
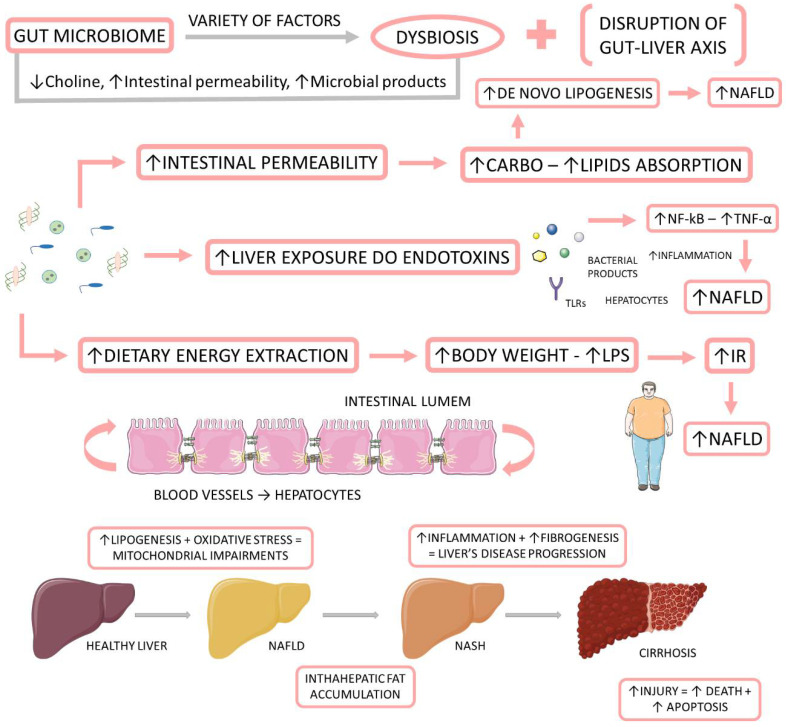
The relationship between the gut microbiome, NAFLD, and NASH. The gut microbiome is affected by a variety of factors to be in dysbiosis. When dysbiotic, the gut microbiome becomes disrupted and starts to cause alterations in the intestinal permeability, leading to augmented liver exposure to endotoxins and dietary energy extraction. These alterations induce an increase in the intrahepatic lipid accumulation, and they induce liver inflammation and fibrosis. IR: insulin resistance; LPS: Lipopolysaccharide; NF-kB: nuclear factor kappa B; NAFLD: non-alcoholic fatty liver disease; NASH: non-alcoholic steatohepatitis; TNF-α: tumor necrosis factor-alpha.

Recently, some authors have joined in favor of changing the name from NAFLD to metabolic (dysfunction) associated with fat liver disease (MAFLD). They believe that the term NAFLD has been described as an exclusionary condition. It exists only when other conditions such as viral hepatitis B and C, autoimmune diseases, or alcohol intake above a certain threshold are absent. However, MAFLD is present in about one fourth of the global population, and it coexists with other liver diseases. Another argument resides in the debate about the safe limit of alcohol intake and the challenge about the application of questionnaires that are faithful to the real consumption of this beverage. The third point is that the new term could simplify the stratification of the disease without the dichotomous classification between NASH and non-NASH. Finally, the authors argue that the new term would consider the heterogeneity of fat liver disease which facilitates the selection of phenotypes for clinical trials as well as therapies. The diagnosis of MAFLD is grounded on the identification of hepatic steatosis by histology, imaging, or blood biomarkers, in association with one of the following three conditions: excess adiposity, presence of pre-diabetes or DM2, or evidence of metabolic dysregulation [39,40,41,42]. Figure 1 shows the relationship between the gut microbiome, NAFLD, and NASH.

### 1.2. Microbiome

The human microbiome refers to the genomic component of organisms (microbiota) that inhabit a specific human body location [43]. More than 30 trillion microorganisms are part of this ecosystem [44]. It is composed mainly of bacteria, but it includes commensal populations of fungi, archaea, and protists as well, covering all three domains (Bacteria, Archaea, and Eukarya), in addition to viruses [45,46]. These microbes reside in the skin, oral cavity, gastrointestinal, respiratory, and genitourinary tracts, accounting for 1–3% of total body weight [47]. Considering the variability between individuals, the intestinal microbiome has a set of 3.3 million different genes, representing a genome 150 times larger than humans’ [48].

**Figure 2:**
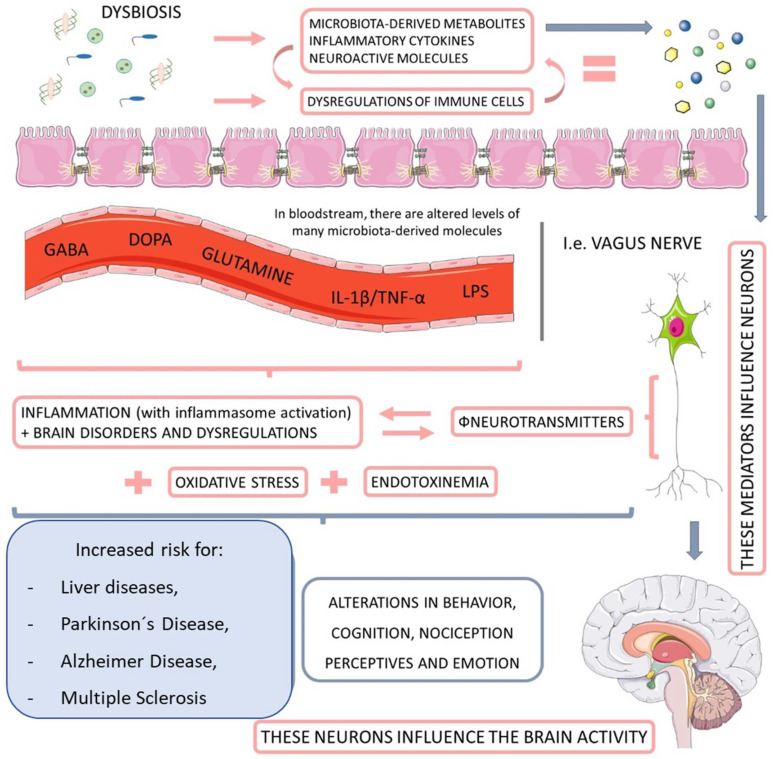
The relationship between microbiota, nervous system, and liver diseases. When dysbiotic, the gut-microbiome-derived metabolites start to cause neuroendocrine dysregulation, principally by impairments in the activity of neurotransmitters. This endotoxemia increases the personal risk for Parkinson’s disease and Alzheimer’s disease, as well as multiple sclerosis and other behavioral and cognitional alterations. GABA: gamma-Aminobutyric Acid; DOPA: dopamine; IL-1ß: interleukin-1β; TNF-α: tumor necrosis factor.

The intestinal microbiota plays a vital role in the metabolism of substrates including carbohydrates, proteins, polyphenols, vitamins, and bile [48,49]. It is closely related to the hosts which develop and tune the immune system [50], as well as protect against pathogenic colonization by competing for fixation sites or nutrient sources, producing bacteriocins (e.g., lactic acid), inhibitory metabolites (short-chain fatty acids and lithocholic acid), stimulating IgA epithelization and mucus production [51,52]. Moreover, an interesting relationship between microbiota and the nervous system has been observed, constituting the brain–gut axis (Figure 2). This extensive communication network connects the gastrointestinal tract with the central nervous system’s cognitive and emotional centers (CNS) [53,54]. 

It was established that the fetus’s intrauterine environment was sterile for a long time, and the microbiome development started during and after birth [55]. However, in the last 15 years, with advances in DNA sequencing technology, this theory has been challenged by scientific evidences that demonstrated the presence of microbes in the placenta, amniotic fluid, the umbilical blood cord, and meconium (even in healthy pregnancies), [55,56,57,58,59,60,61] in addition to the possibility of participation of maternal microbiota and its metabolites in fetal development, inciting the theory of in utero colonization [62].

During and after birth, the newborn is surrounded by many microorganisms. Thus, the development and shaping of the initial microbiota will depend on and vary according to factors such as gestational time, mode of delivery, method of infant feeding, intrapartum, and neonatal antibiotic courses [63]. Neonates born from normal births are likely to have fecal microbiota resembling vaginal microbiota, dominated by *Prevotella* spp. and *Lactobacillus*, while babies born by cesarean section acquire bacteria derived from the hospital environment and maternal skin, such as *Staphylococcus*, *Corynebacterium*, *Propionibacterium* spp. [64]. This differentiation is relevant as neonates born by cesarean section are more susceptible to developing asthma, rhinitis, food allergy, celiac disease, and overweightness over the years [65]. The mode of infant feeding has a substantial influence because breast milk is rich in human milk oligosaccharides (HMOs) which are involved in pathogenic protection, maturation of the intestinal microbiome, and promotion of intestinal barrier function and maturation of immune cells [66]. *Bifidobacterium* bacteria are the most related to the positive effects of HMOs and are generally the most abundant among taxons found in infant intestinal microbiota (up to 90%) [67] that have their production two times higher in breastfed newborns compared to feed formulas [68]. Gestation time is also essential, as premature neonates’ immature intestines may have peristalsis, poor barrier functions, and immunity, which may precede the onset of infection and the inflammatory process by colonization pathogenic bacteria [69]. Although decreasing the colonization of pathogenic bacteria, intrapartum antibiotics administration was also related to the development of necrotizing enterocolitis (NEC). Besides, antibiotic administration in the first six months was correlated with an increased likelihood of developing asthma and obesity [70]. Other factors such as family environment, geographical, and cultural traditions are also documented as influencing infant microbiota [63]. 

In summary, the first colonizers of the newborn’s intestinal microbiota are usually facultative anaerobes, followed by the accumulation of obligatory anaerobes, including *Bifidobacterium*, *Bacteroides*, and *Clostridium* for the following six months [71]. With weaning and the introduction of solid foods from six months onwards, intestinal microbiota diversity increases, with *Actinobacteria* and *Proteobacteria* becoming the dominant components of infant microbiota [72]. At the age of 2.5, the infant microbiota’s composition, diversity, and functional capabilities resemble adult microbiota [73]. This intestinal community will undergo subtle changes until middle age (around 40 years of age), which is a time of relative stability [74]. It has a robust interpersonal character but is generally dominated by Firmicutes and Bacteroidetes, representing up to 90% of its composition and *Actinobacteria*, *Proteobacteria*, and *Verrucomicrobia phyla* [75].

However, even in stable chronological periods, the microbiota is subject to conditions capable of destabilizing homeostasis with the host. This process is called dysbiosis, a compositional and functional change in the microbiota that is driven by a set of factors that disturb the microbial ecosystem to a certain extent that exceeds its capacity for resistance and resilience. Several factors are associated with this phenomenon, including infections, diet, xenobiotics, genetics, familial transmission, circadian disruption, high-fat maternal diet, pregnancy, and physical injury [76]. 

An ecosystem in dysbiosis can damage the host immune system through various mechanisms that collectively stabilize the dysbiotic configuration. These mechanisms include modulation of inflammatory signaling by microbial metabolites, modulation of Toll-like receptor signaling (TLR), and degradation of IgA secreting agent (sIgA). The result is an intestinal epithelium more susceptible to pathobionts and disruption of the immune system, which decreases the protective capacity of the intestinal barrier, stimulating local or systemic inflammatory and immune-mediated processes [76,77,78]. Therefore, the genesis of several diseases had already been related to the microbiota’s dysbiotic configuration, including DM2, obesity, inflammatory bowel disease, cardiovascular diseases, autoimmune diseases, neurodegenerative diseases, NAFLD and its progressive form, non-alcoholic steatohepatitis (NASH) [79,80,81,82,83,84,85].

### 1.3. Microbiome, NAFLD, and NASH

#### 1.3.1. Associations between the Gut Microbiome and NAFLD and NASH

The reciprocal interaction between the microbiome and the liver is established through the vascular route of the portal vein that takes to the liver gut-derived products and the liver feedback route of bile and antibody secretion to the intestine [15]. The liver is first exposed to gut-derived toxic factors, including bacteria, damaged metabolites, or bacterial products (LPS and bacteria DNA) [86]. 

Le roy et al. [87] demonstrated that when exposed to a high-fat diet, germ-free (GF) rats that received a transfer of gut microbiota from hyperglycaemic rats developed fasting hyperglycemia, hyperinsulinemia, and hepatic macrovesicular steatosis. In contrast, GF rats that received a transfer from normoglycemic rats remained normoglycaemic and without steatosis. Pyrosequencing of the 16S ribosomal RNA genes showed that hyperglycemic and normoglycemic rats had distinct gut microbiota regarding phylum, genus, and species. Henao-Mejia et al. [88] showed that sharing the microbiota through coprophagy from mice prone to developing NASH due to modifications in the inflammasome pathway in wild mice led to the development of steatosis and inflammation in the latter group.

Interestingly, some studies have observed that each stage of NAFLD corresponds to a pattern of gut microbiota [89]. A prospective study demonstrated that specific bacterial metagenomic signatures in the gut microbiome of NAFLD patients are a robust predictor of advanced fibrosis in humans. Some species were associated with NAFLD, with the abundance of bacterial species, such as *Proteobacteria*, *Enterobacteria*, *Escherichia*, and *Bacteroides*, being higher in NASH patients compared to matched healthy subjects [90].

There are several mechanisms by which the intestinal microbiota interfere in the progression of NAFLD and NASH. The increase in intestinal permeability, the translocation of dysbiotic bacteria, and the production of metabolites can be associated due to this dysbiotic state, and it is capable of generating disordered inflammatory responses which influence liver metabolism [91].

A new therapeutic attempt that has recently emerged is fecal microbiota transplantation. In this regard, Craven et al. [92] compared two groups: one received an autologous FMT, and the other received allogeneic FMT sourced from three lean, healthy individuals. At the end of the research, there was a significant decrease in the small intestinal permeability of the allogeneic group compared with the autologous group. Although the study did not show changes on biomarkers, it is known that increased intestinal permeability represents a central mechanism behind diseases such as inflammatory bowel disease, systemic inflammation, infection, MS, and NAFLD.

#### 1.3.2. Microbiota-Derived Metabolites and Their Impact on NAFLD and NASH

##### Short-Chain Fatty Acids (SCFAs)

The short-chain fatty acids (SCFA) are the primary end products of fermentation of nondigestible carbohydrates (NDC) that become available to the gut microbiota. The main NDC are acetate, propionate, and butyrate [93,94]. Bacteroides are the main producers of propionate and acetate, while Firmicutes are the primary producers of butyrate [95]. Butyrate and propionate are well documented as gut inflammation relievers [96]. In rats, acetate and propionate supplementation decreased lipogenesis and fat accumulation, shielding them from high-fat (HF) diet-induced weight gain [97]. Svegliati-Baroni et al. [98] reported that the expression of the glucagon-like peptide-1 receptor (GLP-1r) is reduced in the hepatocytes of rats fed diet HF and patients with NASH, and that the activation of GLP-1r in the hepatocytes increased the oxidation of β-fatty acids and improved the insulin sensitivity. In addition, butyrate in particular is able to improve the function of tight junctions and stimulate mucin production, which helps maintain the integrity of the intestinal wall and prevent translocation of bacteria and its products, such as LPS, into the portal circulation [99,100].

##### Bile Acids

Bile acids are molecules produced in the liver from cholesterol and stored in the gallbladder. In addition to facilitating the absorption of lipids, they also play a role in glucose metabolism. The intestinal microbiota converts primary bile acids, including cholic acid (CA) and chenodeoxycholic acid (CDCA) in the distal small intestine and colon of humans into more than twenty different secondary bile acids, such as deoxycholic acid (DCA), lithocholic acid (LCA) and ursodeoxycholic acid (UDCA) [101,102]. Bile acids are indirectly involved in antimicrobial defense mediated by the farnesoid X receptor (FXR). Activation of this receptor reduces fatty acid and triglyceride synthesis in the liver by decreasing the expression of LXR and SREBP-1C [103]. FXR-deficient mice show reduced insulin sensitivity and decreased glucose tolerance [104]. In contrast, FXR activation by selective agonists suppresses bile acid and fatty acid production and increases glucose and insulin sensitivity in obese and diabetic mice. FXR activation also appears to attenuate primary biliary cirrhosis and NASH by reducing bile acid pool and liver fibrosis [105,106]. Bile acids are also closely related to another receptor, Takeda-G-protein-receptor-5 (TGR5). In the intestines, activation of TGR5 on L cells increases secretion of GLP-1, which binds to its receptor located on pancreatic beta cells, raising insulin secretion and reducing glucagon synthesis. The TGR5 is also able to modulate inflammatory processes. Its binding to the receptor reduces the release of pro-inflammatory cytokines by macrophages through the inhibition of NF-kB. Furthermore, in an experimental animal model, TGR5 knockout (TGR5-/-) mice have been shown to display accelerated LPS-induced inflammation in the liver and to suppress the inhibitory effect of TGR5 agonist on the expression of inflammatory mediators when compared with wild-type mice [107].

##### Choline and Trimethylamine

Choline is a constituent of the cells and mitochondrial membranes and the neurotransmitter acetylcholine. Choline-deficient diets have long been used to examine the mechanisms of fatty liver disease and its progression. They reproduce many of the phenotypes seen in humans with NAFLD, including an accumulation of triglycerides in the liver [108]. The phosphorylation of choline for the production of phospholipids and its oxidation as a methyl group donor are the main destinations of this nutrient [109]. Phosphatidylcholine is one of the most important choline metabolites. Its function is related to the packaging and export of triglycerides in very-low-density lipoprotein (VLDL) and to the solubilization of bile acids for excretion [110,111]. The lack of choline alters mitochondrial membranes, decreasing the concentration of phosphatidylethanolamine and phosphatidylcholine, which leads to a decrease in an action potential. This process consequently decreases ATP production and beta-oxidation, causing further hepatic steatosis [112,113]. For example, Arao et al. [114] used a methionine/choline-deficient diet to establish a NASH model and found that mitochondrial DNA content was decreased. The gut microbiome actively metabolizes choline, which may alter its bioavailability and potentially predispose one to choline deficiency [115]. The intestinal microbiota promotes the conversion of choline into trimethylamine, which, upon entering the circulation, will be converted into trimethylamine N-oxide in the liver [116]. Increased production of this substance results in a decrease in choline and consequently in the export of hepatic very-low-density lipoproteins and modulation of bile acid synthesis, which has detrimental effects on the liver, such as increased hepatic fat deposition and inflammatory and oxidative lesions and decreased glucose metabolism [89,117]. 

##### Ethanol

**Figure 3:**
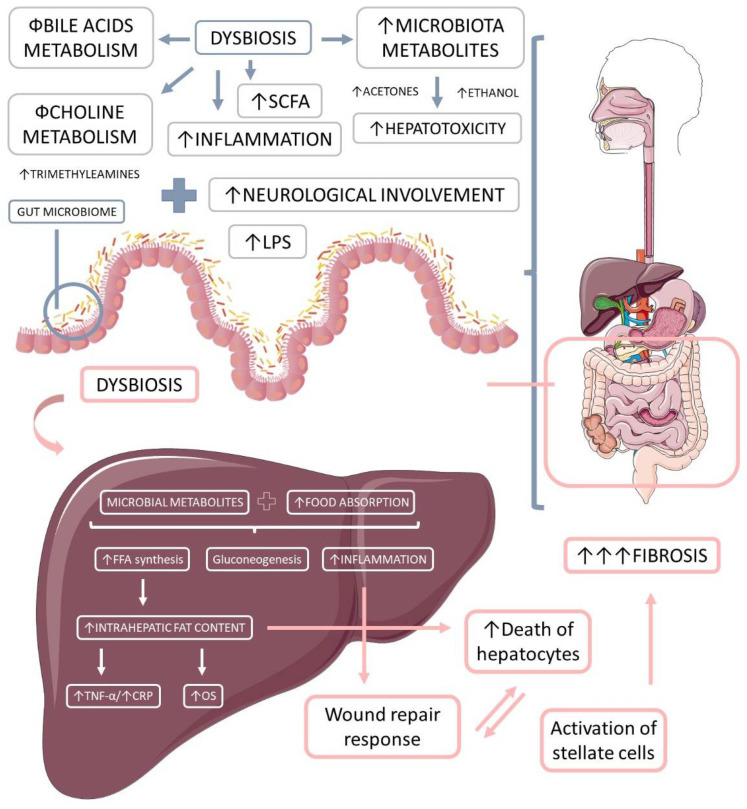
The microbiota-derived metabolites and their impact on liver inflammation, oxidative stress, and the development of liver diseases. Gut microbiome dysbiosis impairs the bile acids and choline metabolism, increases hepatotoxicity, and promotes inflammation. The microbiota-derived metabolites and the augmented food absorptions increase the intrahepatic production and accumulation of lipids, which causes increased inflammation and oxidative stress. Due to these events, the liver loses its capacity for wound repair response, and in addition to the augmented hepatocytes death and the augmented activation of stellate cells, liver massive fibrosis occurs. LPS: lipopolysaccharide; OS: oxidative stress, TNF-α: tumor necrosis factor-alpha; FFA: free fat acids; CRP: C reactive protein.

Ethanol is a microbial metabolite derived from saccharolytic fermentation. As late as 2000, Cope et al. [118] suggested that blood levels of ethanol were related to changes in the gut microbiota. Further, other studies have shown that dysbiosis in NASH patients involves ethanol-producing bacteria such as *Escherchia coli*, *Bacteroides*, *Bifidobacterium*, *Clostridium* [119,120]. One survey found a high increase in ethanol levels in NASH patients compared to healthy individuals or obese non-NASH patients [119]. Gut-bacteria-derived ethanol and its oxidized metabolite, acetaldehyde, are possibly involved in the progression of NAFLD through direct toxic effects on liver cells, through damage to the intestinal barrier generating increased portal endotoxemia, and through upregulation of nuclear factor-κB (NF-κB) signaling inflammatory pathways in peripheral cells [121,122]. Figure 3 shows the microbiota-derived metabolites and their impact on NAFLD and NASH.

## 2. Materials and Methods

### 2.1. Focused Question

This review was built to answer the focused question: Can probiotics or prebiotics interfere with NAFLD or NASH?

### 2.2. Language

Only studies in English were selected.

### 2.3. Databases

**Figure 4:**
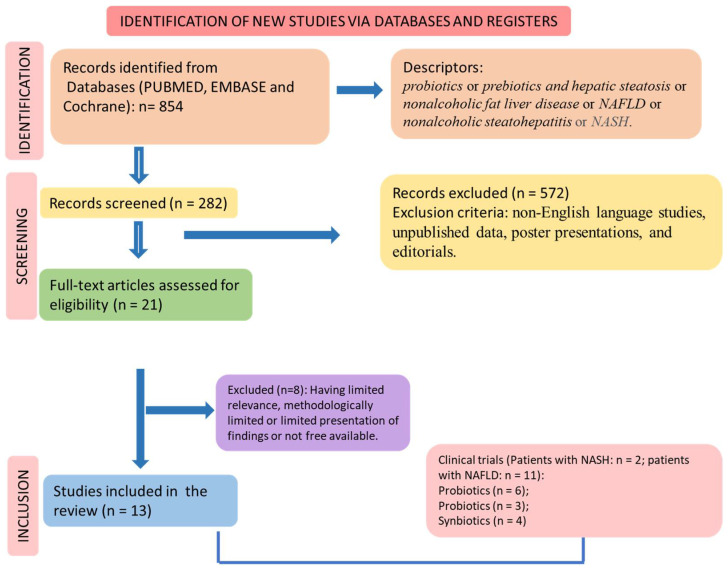
Flow diagram showing the study selection.

This review included studies published in the following databases: MEDLINE–PubMed (National Library of Medicine, National Institutes of Health), EMBASE, and Cochrane. The descriptors were “probiotics or prebiotics and hepatic steatosis or nonalcoholic fat liver disease or NAFLD or nonalcoholic steatohepatitis or NASH”. The use of these descriptors helped us to identify the trials involving the microbiota and NAFLD. The Preferred Reporting Items for Systematic Reviews and Meta-Analysis (PRISMA) guidelines [29,30] were followed, and the flow diagram is shown in Figure 4.

### 2.4. Study Selection

The inclusion criteria were RCTs, primary, and interventional studies, and the exclusion criteria were reviews, studies not in English, editorials, and case reports.

### 2.5. Eligible Criteria

This systematic review’s eligible criteria followed the *PICO* (population, intervention, comparison, and outcomes) format, and the studies involving NASH and microbiome were included. 

### 2.6. Data Extraction

Two independent judges, RC and SMB, independently performed the search to identify the trials in the databases. The abstracts of the studies were evaluated, and full-text articles were also considered to support the decision-making process. Disagreements between the judges were evaluated and decided by another reviewer (LFL). Our review was limited to trials published in the last five years.

The risk of bias in the included trials was evaluated according to each study’s detection, selection, and reporting biases. Moreover, other risks, including patients, interventions, investigation of outcomes, and missing events/data were also considered. The assessment of the biases was performed according to the Cochrane Handbook for Systematic Reviews of Interventions to achieve this quality assessment [123].

## 3. Results

The selection of the included clinical trials is shown in Figure 4 and Table 1. Table 2 brings the risk of bias for the included studies. These trials (n = 13) included 947 patients with 18 to 80 years old. Most of them were double-blind studies. Six studies used probiotic intervention [124,125,126,127,128,129]; three used prebiotics [130,131,132] and six used synbiotics [133,134,135,136]. Only two trials were performed with patients with NASH [124,131].

The clinical trials selected for this review demonstrated that gut microbiota interventions could improve a wide range of markers of inflammation (Lipopolysaccharide (LPS), tumor necrosis factor-α (TNF-α), Interleukin (IL)-6) [125,135], liver injury (alanine aminotransferase (ALT), aspartate aminotransferase (AST), γ-glutamyltransferase (GGT)) [124,125,132,134,136]; dyslipidemia (total cholesterol, low-density lipoprotein (LDL), and triglycerides) [124,125,126,127,134]; obesity (body mass index (BMI), body weight, waist circumference) [124,126,127], and insulin sensitivity (HOMA-IR (homeostasis model assessment of insulin resistance), fasting blood glucose and Vaspin adipokine) [132]. In addition, we noted a decrease in scores used to assess NAFLD and NASH, such as hepatic, fatty liver index (FLI) [125], NAFLD fibrosis, and steatosis scores [131,133,135] and NAFLD activity score [131]. In general, the results of the trials showed that the use of probiotics can reduce BMI, total fat percentage, total cholesterol, triglycerides, fasting insulin, LPS, HOMA, AST, ALT, GGT, TNF-α, IL-6, liver stiffness, fat fraction, fat liver index, vaspin, and Clostridia and Erysipelotrichia classes. Moreover, they can increase the levels of superoxide dismutase (SOD) and glutathione peroxidase (GSH-Px). The use of prebiotics could reduce intra-hepatocellular lipids (IHCL), NASH score, *Roseburia* and *Dialister*, and it can increase *Bifidobacterium* levels. The consumption of synbiotics can reduced BMI, AST, ALT, GGT, TNF-α, NAFLD fibrosis score, and liver stiffness. On the other hand, they can improve *Bifidobacterium* levels.

## 4. Discussion

### The Relationship between Microbiota Interventions and NAFLD and NASH

The included studies in this review evaluated the functionality of interventions on gut microbiota that reflect improvement in markers of NAFLD and NASH. A total of 947 subjects from 13 randomized controlled trials (RCTs) were included. Below, we first discuss the studies performed with probiotics followed by those performed with prebiotics and synbiotics.

Manzhali et al. [124] used a probiotic containing *Lactobacillus casei*, *L. rhamnosus*, *L. bulgaris*, *Bifidobacterium longum*, and *Streptococcus thermophilus* (108 bacteria/capsule in total) as well as fructooligosaccharides for 12 months. All patients from the experimental group (EG) and control group (CG) were advised to maintain a low-calorie diet. At the end of the trial, the CG showed a significant reduction (*p* < 0.05) in TC and BMI compared with baseline, while the EG showed a significant reduction in TC, BMI, AST, ALT, and liver stiffness when compared to baseline and endline. The decrease in BMI in the EG was significantly greater than in the CG. Changes in the microbiota were observed with a significant increase in bacterial strains towards a normal pattern, which did not occur with pathogenic Enterobacteriaceae. At the end of the intervention, the EG had more participants with bacterial strains within their respective normal values in the microbiota when compared to the CG. The bacterial strains with a statistically significant difference were *Bifidobacteria*, *Lactobaccillus*, *E. coli* with normal properties, *Enterococcus feacalis*, nonpathogenic *Staphylococcus*, *Staphylococcus aureus*, *Klebsiella*, *Proteus*, *Citrobacter*, and Enterohemorrhagic *E. coli*. The primary limitations of this study include the fact that the study was nonblinded and that the diagnosis of NASH was not made by liver biopsy (the gold standard).

In the study by Kobyliak et al. [125], 58 participants with NAFLD and diagnosed with DM2 were analyzed. The group that received the multi-probiotic “Symbiter” (concentrated biomass of 14 probiotic bacteria genera *Bifidobacterium*, *Lactobacillus*, *Lactococcus, Propionibacterium*, and *Acetobacter*) for eight weeks showed significant improvement (*p* < 0.05) in markers such as AST, IL-6, TNF-α, fat liver stifness, GGT, and LDL compared to the placebo group. These results are interesting because the study had a relatively short intervention time (two months), with few side effects. Some limitations of this study are that the authors used the US technique instead of biopsy for the diagnostic of NAFLD. Moreover, the short-term follow-up, the small sample size, and the use of metformin could lead to bias in the interpretation of the results. The authors also did not inform the number of men and women included in the study.

Following a strict methodology and with a 12-month follow-up, Ahn et al. [126] investigated the effects of the use of synbiotics on visceral fat area (VFA) and intrahepatic fat fraction (IHF) in NAFLD patients. The synbiotic was composed of six different strains of bacteria (*Lactobacillus acidophilus*, *L. rhamnosus*, *L. paracasei*, *Pediococcus pentosaceus*, *Bifidobacterium lactis*, and *B. breve*). The researchers noted an intense modulation of the microbiota with an increase in specific lineages. There was a significant reduction in body weight, triglycerides, and IHF fraction after 12 weeks in the treated group but not in the placebo group. Interestingly, Agathobaculum, Dorea (OTU 527923), Dorea (OTU 195044), Blautia, Ruminococcus, and Dorea (OTU 470168) were directly related to the decrease in hepatic fat fraction (IHF) and *Eubacterium*; Fusicatenibacter, Dorea (OTU 195044), *Oscillibacter* and *Faecalibacterium* were related to the reduction of BMI. The results show that there is more than a direct action of probiotic strains on the disease; the symbiotic is able to return the microbiota to a more functional and healthier eubiotic state through the growth of other strains. Furthermore, the probiotic group showed significant improvement in inflammatory markers such as IL-6 and TNF-α. The limitations of this trial are the small sample size and the short length of intervention which may not be sufficient to evaluate liver fibrosis. Moreover, the measurements used to assess liver fat content and modulation of inflammation may have dubious results.

Chen et al. [127] conducted a randomized clinical trial involving only obese women because this population has a higher risk factor for MS. The study compared the impact of a diet with 220 g of yogurt and another with 200 g of milk for 24 weeks. The group that consumed the yogurt showed a statistically significant decrease in fat mass, lipid accumulation product, HOMA-IR, fasting insulin AST, intrahepatic lipid, hepatic fat fraction, LPS, fibroblast growth factor, total cholesterol, triglycerides, and biomarkers of inflammation and oxidative stress. Furthermore, yogurt altered gut microbiota composition. The authors concluded that these positive results can be related to improving lipid metabolism as well as reducing inflammation and oxidative stress. Possible biases for this study are that only Chinese women were included, there was a lack of liver biopsy and adipose tissue, and, due to the inclusion of only Chinese participants, other trials are necessary to investigate the effects of yogurt in other ethnic groups.

Another study investigated the effects of multi-strains consisting of Lactobacillus acidophilus, Lactobacillus casei, Lactobacillus lactis, Bifidobacterium bifidum, Bifidobacterium infantis, and Bifidobacterium longum on hepatic steatosis, fibrosis, and biochemical parameters of patients with NAFLD. Although the authors did not find differences between the probiotic group and the control in the above parameters, they found that the use of probiotics can stabilize the mucosal immune function and could protect NAFLD subjects against augmented intestinal permeability. Nevertheless, the sample size of the clinical trial was small, and the participants of the probiotic group reduced the fat intake during the study, which could affect the final results of the study [128].

Chambers et al. [130] designed a double-blind study in which they compared two groups. Participants received either 20 g/d of inulin control or inulin-propionate ester (IPE) for 42 days. At the end of the follow-up, IHCL significantly increased within the inulin-control group (*p* = 0.012), not observed in the inulin-propionate ester group (*p* = 0.635). According to the result, the inulin-propionate ester supplementation did not reduce liver fat content but apparently attenuated the increase in liver fat caused by inulin supplementation. As for the weaknesses of the study, the small number of participants (n = 18) and the fact that they accepted a wide BMI range (20–40) kg/m^2^ may have compromised the results. It is suggested that, in hepatocytes, propionate is able to compete with acetate, the main product of inulin metabolism and which is involved in de novo lipogenesis (DNL), thus decreasing fat accumulation in the liver.

In a placebo-controlled, randomized pilot trial, 14 participants were divided in two groups in which they received either isocaloric placebo for 36 weeks, and oligofructose (8 g/day for 12 weeks followed by 16 g/day for 24 weeks). The prebiotic group showed a statistically significant reduction in hepatic steatosis (*p* < 0.05) and in NAFLD Activity Score (NAS) (*p* < 0.05) compared to the placebo group. Over 36 weeks, the prebiotic group increased *Bifidobacterium* spp. abundance (*p* = 0.017) and reduced *Clostridium* cluster XI (*p* = 0.030) relative to placebo. The study’s main limitations are the small number of participants (n = 14) and the absence of double-blind testing. On the other hand, this study included only patients with NASH diagnosed by liver biopsy, the gold-standard method [131]. 

Chong et al. [132] conducted a 12-week double-blind, placebo-controlled, parallel three-arm trial with 62 participants. Sixty participants were randomized into three groups: 400 mg metronidazole twice daily in Week 1 and then 4 g inulin twice daily, placebo twice daily in Week 1 then inulin, and placebo–placebo. Before receiving the investigational product, all 62 participants underwent a very low-calorie diet for four weeks. After the diet, the participants were followed for another 12 months, taking the investigational products. When comparing the metronidazole group with the placebo–placebo group, the researchers found significant decreases (*p* < 0.05) in ALT and AST. The microbiota after the diet was collected and analyzed, and it showed a reduction in the genus *Roseburia, Streptococcus*, and *Diallister*. The study did not present the intestinal microbiota profile after the use of the medications, due to lack of collection, which decreased the potential results of the research. 

In a trial using synbiotic yogurt consumption, the authors showed improvement in hepatic steatosis and liver enzymes in patients with NAFLD. This interesting study evaluated the consumption of a synbiotic or a conventional yogurt with 1.4% fat. In both, starter cultures of *Lactobacillus delbrueckii* subsp. *Bulgaricus* and *Streptococcus thermophilus* were used. The synbiotic yogurt also had *Bifidobacterium animalis* subsp. *lactis* as a probiotic and inulin (1.5 g) as a prebiotic. However, this trial had a relevant bias; there was a difference in smell and taste between the synbiotic and the conventional yogurt, making blinding of yogurts not possible [136].

Scorletti et al. [133] conducted a double-blind phase 2 placebo-controlled trial including participants with NAFLD. The patients were assigned to groups that received the synbiotic agents (fructooligosaccharides, 4 g twice per day and *Bifidobacterium animalis* subsp. lactis BB-12) or placebo for 10–14 months. The results showed modifications in the fecal microbiomes, but no reductions in liver fat content or markers of liver fibrosis were observed. Although the authors used a rigorous design and long duration (12 months) for the study, one important limitation was the use of a symbiotic composed of only one strain of bacteria (*Bifidobacterium animalis* subspecies *lactis*) and the evaluation of outcomes of the intervention on modifications in gut microbiota using an ex vivo model performed to mimic the human colon. 

The study of Behrouz et al. [134] investigated the use of probiotics and prebiotics in patients with NAFLD and observed reductions in AST, ALT, triglycerides, and total cholesterol but did not observe modifications in biomarkers of inflammation such as C Reactive Protein. However, some limitations should be mentioned in this study. The authors allude to the possibility of adverse effects but did not show whether or not they existed. Moreover, the trial had a relatively short duration, and it is not possible to guarantee that the metabolic parameters reduction would be sustained. They also did not use liver fibrosis to investigate the severity and progression of liver disease.

Abhari et al. [135] investigated the effects of using spores of *Bacillus coagulans* (GBI-30) and inulin in NAFLD patients that were assigned to receive a synbiotic or a placebo capsule for three months. They observed a significant reduction in AST and ALT in the treated group. Moreover, they found a significant decrease in tumor necrosis factor-α, nuclear factor-kB, and hepatic steatosis. However, the supplementation had no effects on cardiovascular risk parameters. The biases of this study were the lack of liver biopsy and investigation of the microbiome that could corroborate the factual findings of the study and the small amount of inulin that could produce no effects on the disease. 

Chong et al. [129] performed a proof-of-concept study on patients with NAFLD. They evaluated several parameters such as systemic inflammation, endothelial function, oxidative stress, insulin resistance, and liver injury. The treatment consisted of taking a supplement named VSL#3^®^, probiotic, or placebo for ten weeks, and measurements of endothelial function, oxidative stress, inflammation, insulin resistance (performing HOMA-IR), and liver conditions (AST, ALT, and fibrosis risk score) were performed before and after the intervention. The authors did not find a significant improvement in cardiovascular risk biomarkers and liver injury. The limitation of this study includes a small sample size and a short follow-up. 

NAFLD is directly associated with insulin resistance, visceral obesity, dyslipidemia, and chronic inflammation. Insulin resistance plays a critical role in the pathophysiology of NAFLD/NASH. Insulin can act in peripheral cells, leading to glucose uptake, storage mechanisms (glucogenesis), and protein and fat synthesis. When these metabolic pathways are activated, there is an inhibition of catabolic processes such as lipolysis, glycogenolysis, and gluconeogenesis. With high consumption of carbohydrates, lipids, and a sedentary lifestyle, the excessive energy consumption is stored in the adipose tissue. Insulin resistance augments the flow of fatty acids to the hepatic cells by boosting hepatic lipogenesis generating an imbalance in metabolism. This imbalance is responsible for the excessive intrahepatic accumulation of lipids, which is lipotoxic, resulting in mitochondrial damage, endoplasmic reticulum stress, and autophagy. The imbalance in the metabolism and visceral fat deposition is also associated with increase in the release of pro-inflammatory cytokines and increase in peroxidation processes. The production of malondialdehyde is also related to perisinusoidal and periportal fibrosis due to the activation of nuclear factor κβ and by regulation of the expression of inflammatory cytokines such as IL-8 and TNF-α. This inflammatory and oxidative scenario is related to the activation of hepatic stellate cells, contributing to fibrosis. The peroxidation of the membrane phospholipids modifies the permeability and leads to ballooning hepatocytes. These histological modifications can result in fibrosis, cirrhosis, hepatocellular carcinoma, and increased mortality from liver causes [38,137,138]

## 5. Conclusions

Although microbiota modulators do not play a healing role, it is possible to evaluate their function as an important adjunct in pathological processes involving NAFLD and its spectrums, either by improving the intestinal barrier, or preventing the formation of toxic metabolites for the liver, or by acting on the immune system. There is a high range of inflammatory biomarkers that may be altered in the disease and that can be attenuated with the use of the therapies. Some clinical trials have shown an increase in bacteria from the phylum Firmicutes, which is related to the formation of butyrate and other SCFAs, which provide energy to colonocytes, maintain the integrity of the intestinal barrier, and thus prevent exposure of toxins to the liver and inordinate inflammatory responses from the immune system. 

The trials included in our review showed that the use of probiotics, prebiotics and synbiotics were related to reduction in BMI, total fat percentage, total cholesterol, triglycerides, fasting insulin, LPS, HOMA, TNF-α, IL-6, liver injury (AST, ALT, liver stiffness, fibrosis index, intracellular lipids, NAFLD and NASH scores), and Clostridia and Erysipelotrichia classes. On the other hand, they can improve *Bifidobacterium* levels. We suggest that more studies with larger sample and increased follow-up should be performed so that physicians can make relevant and effective choices for the use of these supplements in the therapeutic approach to NAFLD and NASH.

## Figures and Tables

**Table 1:** Descriptive table of the included studies.

Reference	Model/Country	Population	Intervention/Comparison	Outcomes	Side Effects
Use of probiotic					
[124]	Randomized, controlled, open label, prospective, multicenter clinical trial/Ukraine.	75 participants, 27 ♂, mean age: 43.9 and NASH diagnosis.	75 patients with NASH fed a low-fat/low-calorie diet were randomly divided into the control group (n = 37) and the experimental group (n = 38). The probiotic cocktail (*Lactobacillus casei, L. rhamnosus, L. bulgaris, Bifidobacterium longum, and Streptococcus thermophilus* (10^8^ bacteria/capsule) and fructooligosaccharides 1 × d for 12 weeks.	The experimental group exhibited a significant reduction (*p* < 0.05) in BMI, TC, TG, ALT, AST, and LS. A significant augment in the microbial community towards the normal range, with the exception of the pathogenic enterobacteria strain, was also observed.	No adverse events were observed.
[125]	Randomized, parallel, double-blind, placebo-controlled, single-center clinical trial/Ukraine.	58 patients, 18–65 y, with BMI ≥ 25 kg/m^2^, DM2, and NAFLD.	Participants were separated into two groups: one received the multi-probiotic “Symbiter” (concentrated biomass of 14 probiotic bacteria genera *Bifidobacterium, Lactobacillus, Lactococcus, Propionibacterium* and *Acetobacter*) 10 mg/day, n = 30 and the other received a placebo (n = 28)/8 w.	After intervention, compared to the placebo group, the probiotic group presented a statistically significant reduction in AST, GGT, LDL, TNF-α, and IL-6. In the probiotic group, FLI significantly decreased. No modifications were seen in the placebo group.	Probiotic group: Diarrhea (n = 1) and mild headache (n = 1). Placebo group: mild abdominal pain (n = 2) and nausea (n = 1).
[126]	Randomized, double-blind, placebo-controlled, and multi-center clinical trial/Korea	65 NAFLD participants (32 ♂, 19–75 y) with BMI ≥ 25 kg/m^2^ and mean hepatic MRI-PDFF value ≥ 5.0%.	Subjects were divided into two groups; one received a probiotic mixture, while the other a dextrin-based placebo/12 m.	The group that received probiotic exhibited a significant reduction in body weight, BMI, right liver FF, left right FF, whole liver FF, total fat mass, total body fat percent (%), visceral fat grade (*p* = 0.0029), cholesterol, IL-6, and TNF-α.	In the placebo group, n = 1 died of interstitial pneumonia.
[127]	Randomized, parallel, controlled, multicenter trial/China.	118 partipants, all female, 36–66 y, with a WC ≥ 90 cm and BMI ≥ 28 kg/m^2^.	100 obese women with NAFLD and MS were randomly divided to consume 220 g/d of either conventional yogurt or milk for 24 w.	Compared with milk, yogurt significantly decreased FM, WC HOMA-IR, fasting insulin, 2-h insulin, 2-h AUC for insulin, ALT, IHL, and hepatic fat fraction, TG, TC, and LAP. Yogurt also significantly decreased serum LPS, FGF21, TNF-α, Vaspin, the relative abundance of the Firmicutes phylum, Clostridia and Erysipelotrichia classes, Clostridiales and Erysipelotrichales orders, Erysipelotrichaceae and Veillonellaceae families, and Blautia, Pseudobutyrivibrio, *Eubacterium ventriosum* group, *Ruminococcus* and *Dialister* genera; and significantly increased the relative abundance of the Negativicutes class and *Phascolarctobacterium* genus.	No adverse events were observed.
[128]	Randomized, double-blind, placebo-controlled trial with NAFLD patients/Malaysia	39 participants (28 ♂, 36–74 y) with NAFLD)	Subjects were supplemented with a probiotic sachet (MCP^®^ BCMC^®^ strains) or a placebo/six months (a multi-strain probiotics (MCP^®^ BCMC^®^ strains) with six different *Lactobacillus* and *Bifidobacterium* species (30 billion CFU were used).	No significant changes were observed for hepatic steatosis, fibrosis inflammation scores, ALT, cholesterol, triglycerides, and fasting glucose.	Patients did not report side effects.
[129]	Randomized, double-blinded, placebo-controlled, proof-of-concept study/United Kingdom	35 patients (28 ♂, 7 ♀, 36–74 y 25–70 y) with BMI = 32.6 ± 5.0 kg/m^2^ and a short duration of NAFLD	Participants were randomly divided to take 2 sachets VSL#3^®^ probiotic supplement or placebo/2 × d/10 weeks.	No signifcant diferences were observed in biomarkers of cardiovascular risk and liver injury but signifcant correlations were seen between sVCAM-1 and hsCRP, and HOMA-IR and AST.	bloating, nausea, genital thrush and perianal rash.
Use of prebiotics					
[130]	Randomized, double-blind placebo-controlled trial/United Kingdom	18 participants (10 ♂, 18–65 y) with NAFLD and BMI of 20 to 40 kg/m^2^.	Subjects received either 20 g/d of inulin control or inulin-propionate ester (IPE) for 42 d. The 20 g dose of IPE provided 14.6 g inulin (and 5.4 g propionate bound) to the diet.	The change in intrahepatocellular lipid (IHCL) following the supplementation period was not different between the groups (*p* = 0.082); however, IHCL significantly increased within the inulin-control group.	NR
[131]	Randomized, placebo-controlled, multicenter trial/Canadá.	14 participants with NASH (8 ♂, > 18 y), BMI > 25 kg/m^2^ (Caucasians) and >23 kg/m^2^ (Asians), history of serum ALT >1.5 × normal upper limit, no changes in lipid-lowering or diabetes medication over previous 3 m.	Subjects were divided into either a treatment group that received oligofructose prebiotic 8 g orally 1 × d/12 w followed by 16 g 1 × d/24 w or an isocaloric maltodextrin placebo (PLA) control.	After 36 weeks, compared with the placebo group, the prebiotic group showed a statistically significant reduction in hepatic steatosis (*p* < 0.05). In the probiotic group, there was a significant reduction in hepatic steatosis (*p* < 0.05) and NASH score (*p* < 0.05). Over 36 w, PRE increased *Bifidobacterium* spp. abundance (*p* = 0.017) and reduced *Clostridium* cluster XI (*p* = 0.030) relative to PLA	NR
[132]	Randomized, double-blind placebo-controlled trial, single-center/New Zealand.	62 participants (31 ♂, 18–75 y) with NAFLD.	All participants underwent a very low-calorie diet (VLCD) for 4 w. The metronidazole and inulin group received metronidazole (400 mg 2 × d/7 d) along with inulin (4 g 2 × d/12 w); the placebo and inulin group (Group PI) received metronidazole-like placebo (2 × d/7 d) along with inulin (4 g 2 × d/12 w); the placebo and inulin placebo group received metronidazole-like placebo (2 × d/7 d) along with inulin-like placebo (containing maltodextrin at 4 g 2 × d/12 weeks).	There was a significant reduction in bacteria of the genus *Roseburia* (*p* = 0.005), *Streptococcus* (*p* = 0.0005), *Dialister* (*p* = 0.032). In relation to the baseline, the VLCD presented a significant difference of clinical parameters. After 12 w compared with the placebo—placebo group, the metronidazole—inulin group presented a significant reduction in ALT (*p* = 0.026) and AST (*p* = 0.006).	No adverse events requiring discontinuation of inulin were reported.
Use of synbiotics					
[136]	Open-label, randomized controlled clinical trial/Iran.	102 participants (50 ♂, mean age of 40 y) with NAFLD.	Participants were assigned to two intervention groups (300 g synbiotic yogurt with 10^8^ colony-forming units *Bifidobacterium animalis*/mL + 1.5 g inulin or conventional yogurt/d) and one control group. The intervention groups were advised to proceed with a healthy lifestyle (diet and exercise). The control group only was told to follow a healthy lifestyle alone/24 weeks.	The grades of NAFLD significantly reduced in the synbiotic group compared with the other groups (*p* < 0.001). There was a significant reduction of AST, ALT, alkaline phosphatase and GGT.	No serious adverse events were observed.
[133]	Randomized, double-blind placebo-controlled trial, multicenter/United Kingdom	104 participants, 65 ♂, 37% diabetic, mean age 50.8 ± 12.6 y, with NAFLD.	Participants were randomly assigned to synbiotic agents (fructo-oligosaccharides, 4 g 2 × d, plus *Bifidobacterium animalis* subsp. *lactis* BB-12; n = 55) or placebo (n = 49) for 10–14 months.	Weight loss was associated with significant improvements in ELF (*p* = 0.039) and NAFLD fibrosis score (*p* = 0.027) and also liver stiffness measurement (*p* = 0.025). Synbiotic group: significant increase in *Bifidobacterium* abundance, *Fecalbacterium*, *Actinobacteriae*. *Oscillibacter* and *Aistipes* genus significantly decreased.	Bloating and flatulence) (n = 1).
[134]	Parallel, randomized, double blind, controlled clinical trial/Iran	111 patients diagnosed with NAFLD (43 ♂, 20–60 y)	Subjects with NAFLD received probiotic capsule + placebo of prebiotic (probiotic group), oligofructose + placebo of probiotic (prebiotic group), or placebo of probiotic + placebo of prebiotic (control group)/12 weeks.	Anthropometric measurements reduced in the three groups, but without significant differences. Probiotic supplementation reduced triglyceride, ALT, AST, GGT, total cholesterol, LDL-c.	NR by the authors.
[135]	Randomized pacebo-controlled, double-blind clinical/Iran	53 participants with NAFLD (25 ♂, 47–59 y)	Participants received a synbiotic capsule with 109 spore of *B. coagulans* (GBI-30) + 0.4 g inulin and lifestyle intervention (diet and exercise)/d/12 weeks.	The use of *B. coagulans* + inulin + lifestyle modifications is superior to only lifestyle modifications to reduce steatosis and and TNF-α in patients with NAFLD. No modifications in cardiovascular risk factors were observed.	Patients did not report side effects.

ALT: alanine aminotransferase, DM2: Type 2 Diabetes mellitus; BMI: body mass index; FF: Fat Fraction; FLI: fatty liver index; FM: fat mass; FGF21: fibroblast growth factor 21; GSH-Px: glutathione peroxidase; GGT: γ-glutamyl transferase; IHCL: intrahepatocellular lipid; IL-6: interleukin 6; IPE: inulin-propionate ester; IR: insulin resistance; HFF: hepatic fat fraction; HOMA-IR: homeostasis model assessment of insulin resistance; LS: liver stiffness; LPS: lipopolysaccharide; MS: metabolic syndrome; NAFLD: non-alcoholic fatty liver disease; NR: not reported PDFF: hepatic proton density fat fraction; SOD: superoxide dismutase; TC: total cholesterol; TG: triglycerides; TNF-α: tumor necrosis factor-α; VLCD: very low-calorie diet; VLCD: very low caloric diet; WC: waist circumference; Vaspin: human visceral adipose-specific serine protease inhibitor.

**Table 2:** Descriptive Table of the Biases of the Included Randomized Clinical Trials.

Study	Question Focus	Allocation Blinding	Double- Blind	Losses (>20%)	Prognostic or Demographic Characteristics	Outcomes	Intention to Treat Analysis	Sample Calculation	Adequate Follow-Up
use of probiotics									
[124]	Yes	No	No	No	Yes	Yes	Yes	No	Yes
[125]	Yes	Yes	Yes	No	No	Yes	Yes	No	Yes
[126]	Yes	Yes	Yes	No	Yes	Yes	Yes	Yes	Yes
[127]	Yes	No	No	Yes	No	Yes	Yes	Yes	Yes
[128]	Yes	Yes	Yes	Yes	Yes	Yes	Yes	Yes	Yes
[129]	Yes	Yes	Yes	Yes	Yes	Yes	Yes	NR	No
use of prebiotics									
[130]	Yes	Yes	Yes	No	Yes	Yes	Yes	No	Yes
[131]	Yes	Yes	Yes	No	Yes	Yes	Yes	No	Yes
[132]	Yes	Yes	Yes	Yes	Yes	Yes	Yes	Yes	Yes
use of synbiotics									
[136]	Yes	No	No	No	Yes	Yes	Yes	Yes	Yes
[133]	Yes	Yes	Yes	Yes	Yes	Yes	Yes	Yes	Yes
[134]	Yes	Yes	Yes	Yes	Yes	Yes	Yes	NR	Yes
[135]	Yes	Yes	Yes	Yes	Yes	Yes	Yes	Yes	Yes

NR: not reported.
